# Developing a Framework to Increase Access to Mental Health Services for Children With Special Needs in Ethiopia

**DOI:** 10.3389/fsoc.2020.583931

**Published:** 2020-12-17

**Authors:** Tammy L. Hughes, Cydney Quinn, Amy Tiberi, Waganesh A. Zeleke

**Affiliations:** Department of Counseling, Psychology and Special Education, Duquesne University, Pittsburgh, PA, United States

**Keywords:** healthcare, well-being, mental health, Ethiopia, children

## Abstract

The availability and accessibility of Westernized mental health diagnostic processes and evidence-based treatments are limited in developing countries, such as Ethiopia (Kakuma et al., 2011; Hohenshil et al., 2013; Wondie, 2014; Zeleke et al., 2017b). Similar to other developing nations, there is (a) a lack of health care services for mental practices to build on, (b) limited services that are well-suited to the culture (Wondie, 2014; Zeleke et al., 2019), (c) limited scientific literature useful for documenting the needs of the Ethiopian public, and (d) too few mental health professional preparation programs (Zeleke et al., 2019). Whereas Western cultures generally follow the biomedical model conceptualization and treatment of disease, non-Western cultures, such as Ethiopia tend to adhere to traditional and religious views to explain the origin of mental illness (Kortmann, 1987; Jacobsson and Merdassa, 1991). Mental health symptoms may be attributed to supernatural causes or other spiritual crises, rather than a combination of biopsychosocial influences. As such, individuals seeking help with mental health symptoms in Ethiopia are mostly limited to family, friends and local community healers (Zeleke et al., 2017a, 2019). When individuals are brought to the few places providing Westernized mental health care, it is often only after exhausting other traditional and religious alternatives (Bekele et al., 2000). Even when there is a desire to seek Westernized services, socioeconomic circumstance, cultural factors (e.g., a focus on collectivism practices), negative attitudes toward mental illness, along with unfamiliarity and fear of these new practices, are noted barriers to receiving treatments. Beliefs passed down through cultural taboos go on to effect multiple generations. Not only do barriers affect individuals, but they also negatively impact the range of services for children, families and communities. With the ultimate goal of improving mental health care access for children, a full appreciation of the context is essential.

## Ethiopian Context Barriers

The prevalence of mental health disorders in Ethiopia is reported to be 18% in adults and 15% for children (Sathiyasusuman, 2011). Like other developing country children's mental health needs are high and evident from early childhood throughout adolescence. In a recent study, mothers reporting perinatal depressive symptoms indicated that they were more comfortable conceptualizing these experiences as distress brought on by an external source, such as poverty, physical ill-health or supernatural factors rather than being mental health related (Molenaar et al., 2020). Authors noted that Westernized explanations of mental health symptomology would be inconsistent with their cultural beliefs. Furthermore, these participants low empowerment status may also contribute to statements that seeking any treatments would be inappropriate for such distress. In another example, Ethiopian parents report limited interest in collaboration with non-traditional professionals for their children. This caution remained even for parents of children acknowledging the need for treatment for their children due to their cultural beliefs (Zeleke et al., 2017a).

It is also noteworthy that Ethiopian service providers too are uncomfortable about the providing Westernized mental health care. First, providers indicate that they have limited training in evidence-based practices (i.e., triaging, diagnosing, screening, and referral) that are prioritized by the West and they have some difficulty translating need for these priorities to families (Zeleke et al., 2017b). Second, they are both reluctant and under prepared for collaboration between Westernized practices and existing community practices (Zeleke et al., 2017b). Third, they are cautious about over promising how new service provisions would bridge the gap between the cultural and biomedical models when in reality there are a limited number of practitioners within the country.

There are four universities in Ethiopia offering comprehensive research and training programs (Langhaug et al., 2020). According to a 2015–2016 study by the National Mental Health Strategy (of Ethiopia), there were 40 practicing psychiatrists, 461 psychiatric nurses, 14 psychologists, and three social workers for 85 million Ethiopians. A recent healthcare workforce estimate shows Ethiopia has a low number of healthcare providers compared to other countries in East Africa (Hanlon et al., 2019). In the predominantly rural area of Ethiopia alone, mental illness comprises 11% of the total burden of diseases, with schizophrenia and depression constituting the top ten most burdensome conditions, out-ranking HIV/AIDS (Federal Democratic Republic of Ethiopia Ministry of Health, 2012, p. 9). While mental health problems constitute ~12% of diseases within Ethiopia (Alem and Kebede, 2006), only about 2% of the health budget within the country is allocated to address mental health concerns (Desta, 2008).

Taken altogether, when considering how to prepare an effective mental health campaign to address both clinicians and the public about the range of intervention and treatments available for Ethiopians, it is important to examine how and when to bridge services. Similarly, it is important to prioritize the positive downstream effects of mental health provisions for children. Increasing treatment accessibility and acceptability will require a long-term joint effort from researchers and practitioners, as well as the central and local government, for mental health care to become a central part of Ethiopian public health that extends to parents, children, and families (Zeleke et al., 2019).

## Traditional and Western Approaches to Mental Health Needs and Treatment

The World Health Organization (WHO) defines health as a state of complete physical, psychological and social well-being that “is not merely in the absence of sickness.” Furthermore, it states that the highest attainable standard of health is one of the fundamental rights of every human being without distinction of race, religion, political belief, economic, or social condition (World Health Organization, 2001). The definition is holistic and growth oriented, and has guided health care provision and development of health care systems across the world since the 1940's. The conceptualization of health, however, is culturally and socially defined.

In Western societies, health is often divided into separate physical and mental categories, where disease is conceptualized as a separate influence. Disease is described as a deviation from the biological norm, rather than a part of the health continuum. In this mindset, care is deduced from symptoms, and treatment is applied to the individual aimed at changing the cause of the symptom. Invariably, whereas biomedicine is the dominant health care system in Western societies, as noted, traditional medicine is often the path patients take in developing countries (Ibeneme et al., 2017) in order to rid the body of disease.

Health and well-being in Ethiopia is best understood as a state of equilibrium among physiological, spiritual, ecological, and social factors surrounding the lives of people (Janetius et al., 2013). Poor health may be a result of an unbalanced state of these pursuits. This holistic understanding of health arose from the traditional view that health is a gift of God and evil forces can deter it. Similar popular beliefs regarding health and sickness include: the presence of supernatural forces that can enter a person's body to disturb the health; the shadows cast by an evil eye could be the sources of sickness; environmental hazards, poor hygiene, and climatic conditions, such as heat, rain, or cold wind could cause illness. Further, psychological well-being is not differentiated from physiological well-being.

In this context, health care is based on an inductive approach (i.e., traditional premises of the cause) and treatments can include adapting to the environment rather than changing it directly as in Western medicine. As such, traditional healers may be the first, and typically the only form of service delivery. Even when more Westernized services are available or accessible, people do not tend to utilize the services (Desalew and Yigzaw, 2007; Zeleke et al., 2019) and will only resort to modern mental health services after trying, and failing, with other traditional healing. In this way, traditional beliefs hindered the development of westernized service provision to those with mental health concerns (Kortmann, 1987; Jacobsson and Merdassa, 1991; Uppal et al., 2014), especially in the rural communities (Janetius et al., 2013).

### Traditions Define What Can Be Healed

Traditional treatments are thought to address a variety of concerns including, conflict within relationships, feelings of anxiety or depression and other impairing psychiatric symptoms (e.g., psychosis). Providing care as a midwife, bone setting, or conducting minor surgeries (i.e., circumcision) are common. Therefore, owing to this traditional viewpoint, people go to priests or healers for each and every sickness—physical, psychological, or emotional (Janetius et al., 2013). Evidence for success are found in family testimonials as well as an emerging literature that seeks to document the ethnobotany of medicinal plants used to treat mental illnesses in both traditional conceptualizations (e.g., evil eye, devil sickness, evil spirit, etc.) and Western conceptualizations (e.g., ADHD, depression, psychosis, etc.) of symptoms (Tesfahuneygn and Gebreegziabher, 2019).

Mental health challenges, such as adjustment disorder, depression, and anxiety are typically not recognized as a psychological problem in traditional cultures. Neurological conditions that begin in childhood, such as ADHD, are often explained as a response subsequent to psychosocial stresses linked to poverty, abuse, and neglect, rather than given an appreciation to the biological contributions of these symptoms. Although some severe psychiatric disturbances in adults loosely correspond to Western conceptualizations, the Ethiopian context recognizes only a portion of the syndromes and symptoms, even less so in children. Examining the presence of autism in Ethiopia revealed that 40.2% of parents attributed the child's diagnosis to a “spirit possession” and 27.5% of the sample attributed the child's symptoms as a result of “a sinful act” (Tilahun et al., 2016). When mental health diagnoses are not understood using Western conceptualizations, there is confusion about the importance of Westernized mental health care, which then limits the willingness to engage in it. For individuals where the onset of symptoms occurs in childhood, this can mean years of suffering that could be improved via Western mental health care. Indeed, evidence based practices used in the United States are shown to positively impact children's developmental disorders (Blueprints for Healthy Youth Development, 2018), such as autism, adjustment disorder, depression, anxiety and trauma related conditions even under economic strains (Reiss, 2013).

### Traditions Define Who Can Provide Healing

Ethiopians tend to be attracted to traditional care for multiple reasons: higher cultural acceptability, relatively lower cost, accessibility, availability, shared social norms, and beliefs about the meanings cause and treatment of illness (Lambert, 2001; World Health Organization, 2001; Yemane et al., 2006; Belachew et al., 2019). Simplicity and convenience, inclusiveness, and positive personal or family experiences are also a motivator (Bannerman et al., 1993; Inter-Agency Standing Committee, 2007; Shih et al., 2010). Word of mouth, advice from friends and families, as well as referral from fellow traditional healers or even modern health care providers promulgate the influence of these healers (Mudimu et al., 2003; Tesfahuneygn and Gebreegziabher, 2019). An individual's belief systems and culturally relevant explanation of illness result in the view that healers have more extensive expertise than biomedical oriented practitioners (Alem et al., 1999; Tesfahuneygn and Gebreegziabher, 2019). Therefore, when considering implementing a mental health service system that differs from their traditional treatments, some may ignore the importance due to their strong beliefs that a traditional healer can provide the appropriate amount of care. Joining, rather than shunning, this care is likely the best path forward (Janetius et al., 2013; Workneh et al., 2020).

### Who Are Traditional Healers?

Typically, traditional healers fall into the categories of faith healers, divine healers, herbalists, and mixed healers (Belachew et al., 2019). Faith healers include religious leaders that use the power of God to heal sickness and typically practice in churches, mosques, or holy water places (Rajendra, 1995). Divine healers include those who engage in ritualists or spiritualists or those who practice astrology or read zodiac signs (also known as *kalechas* and *tenquay/wizards*). Faith healers and divine healers can also be known as spiritual healers. Spiritual healers are those who are identified to have magical power, usually governed by either a bad spirit to make a person ill or a good spirit to protect from developing a mental illness. These individuals exorcize malign spirits, such as *buda* (evil eye) and *ganen* (devil) by the use of incantation, sorcery, enchantment, exorcism, and certain rituals (Alem et al., 1999). Aside from spiritual healers, herbalists use herbs, plant remedies, or extracts of animals to heal individuals and mixed healers use both rituals and herbal medicine (Belachew et al., 2019). Lastly, there are also secular healers who are involved in manipulation of the body using a variety of techniques, such as bone-setting by the orthopedic surgeon (*wegesha*), assisting births by a traditional midwife (*Yelimd awalaj)*, dressing wounds or excising affected body parts, pulling out “bad teeth,” or cutting out uvula or tonsils. These traditional healers are an integral part of the society and the people, and are believed to share similar beliefs and attitudes toward all health events that occur in life (World Health Organization, 2001).

When modern medicines and therapies are presented as an updated version or an extension of traditional healing practice there have been a greater acceptance to changes in service delivery (Janetius et al., 2013; Yemataw and Mastewal, 2019). For example, as mental health service providers (e.g., counselors, psychotherapists, social workers) slowly introduce Western models of counseling and psychotherapy and that are consistent with traditional Ethiopian healing practices families have begun to replace or supplement traditional healers (Yemataw and Mastewal, 2019; Zeleke et al., 2019). Active listening, empathy, and person-centered approaches found in traditional healings are also central to Western therapist-client relationship (Zeleke et al., 2019). Community supports evident in westernized social worker practice, can be seen as similar to the popular practice among Ethiopian Orthodox Christians called Yenesha Abat—which involves a priest serving as a family mentor or guide who makes frequent visits to the home. Again, these similarities appear to be a promising link from a traditional healing relationship to a westernized therapeutic service (Zeleke et al., 2019; Molenaar et al., 2020).

### Ethiopia's Move Toward Mental Health Modernization

In 2012, with 80% of the population using traditional healing practices (Yemataw and Mastewal, 2019) the Government of Ethiopia developed and implemented the Ethiopian National Mental Health Strategy (Federal Democratic Republic of Ethiopia Ministry of Health, 2012). The strategy described goals in training mental health professionals and a desired outcome to increase effective mental healthcare delivered via primary healthcare settings. To date, most of the gains have occurred in trainings for psychiatrists, psychologists, and social workers that were subsequent to updates in professional preparation at the University of Gondar and Addis Ababa University (Wondie, 2014). In partnership, the Federal Ministry of Health also worked with WHO and the European Union and Foundation d'Harcourt to successfully implement the Mental Health Gap Action Programme (mhGAP) which aims to expand and scale up services for mental, neurological, and substance use disorders (World Health Organization, 2016). Although some increases to mental health services have begun to spread to in cities and urban centers, counseling and psychotherapy for the individual has not yet deep-rooted in Ethiopia (Janetius et al., 2013).

Alongside the country's accelerating economic and social development, however, there has been a growing acceptance of the need for *societal* mental health in Ethiopia (Lund et al., 2012; Wondie, 2014). The shift to prioritizing the collective, rather than individual, need has allowed for an acceptance of modern services. Furthermore, it appears that promoting the positive effects of a healthy family, particularly as it relates to mental health, has provided an opening toward discussions about mental well-being which is more consistent with Ethiopian health mindset. At the same time, data shows Ethiopians have been accessing their primary care physicians at an increasing rate in both rural and urban communities. With these facts in mind, there was a determination to integrate mental health and well-being care into the primary care settings already established within communities (Wondie, 2014) and in the urban-rural partnership (Workneh et al., 2020). By toggling mental care with primary healthcare, mental health care service providers could support prevention health and well-being efforts as well as treatments for acute issues. Mental health care could now include screening for symptoms, triage issues for urgency, and identifying the best approach (i.e., individually or collectively) for interventions (Zeleke et al., 2019). Placement of mental health services in primary care also opens up possibility to expand services to address child and family concerns.

## Call for Collaborative Efforts

To expand and strengthen the capacity of the overall functioning of the mental health care system and the delivery of services sensitive to Ethiopian culture, a comprehensive effort is required. At the micro-level, mental health care focuses addressing the needs of individuals including conceptualizations about the cause or origin of symptoms, delivery of a range of treatments for management of distress, as well as when to move between traditional and western practices. Meso-level priorities include monitoring of services, advocacy for expanding or targeted care and evaluation of the implementation of programs offerings provided to meet local population needs. Macro-level work requires careful attention to national level policy making, responding to advocacy requests and developing the relationship between research, professional preparation and public health initiatives. In Western cultures, individuals are familiar with addressing concerns across these levels resulting in benefits, such as: improved knowledge about the range of mental health services offered, improved attitudes and better understanding of patient's needs by service providers, and better access to and acceptability of health care services for all members of the community including children.

However, even as new primary care opportunities look promising, it is clear that Ethiopia faces many challenges (Workneh et al., 2020) related to the collaboration and integration of resources across the micro-, meso-, and macro- levels (Abayneh et al., 2020). The most obvious examples are that there is limited government support and push in, limited policies supported by funding, lack of education and training opportunities—and the ever present stigma related to mental health problems—all resulting in individuals experiencing disability, illnesses, premature mortality rates, and human rights abuses, among other challenges (Abayneh et al., 2020). The following suggestions are recommended to systematically strengthen the mental health care system and delivery of services in Ethiopia.

**Determine local needs (e.g., maternal and child health, perinatal distress, HIV, etc.) and resources to guide practice and policy**. The Ethiopian public and government need researchers who can provide them with updated information on how to best support their mental health care (Langhaug et al., 2020). Officials need to understand how research as an integral part of their country's development, help to destigmatize mental health views, and support evidence-based interventions that can support the mental health needs for the people they govern (Langhaug et al., 2020).
° *Provide funds for universities to engage in research*. Currently universities in Ethiopia place more weight on teaching, rather than on finding professors who will allot their time to research and scholarship (Langhaug et al., 2020). Projects related to all aspects of the mental and medical health care system should be prioritized (e.g., micro-, meso-, and macro-levels).
° *Increase Government Participation*. Without guiding policies, dedicated government effort, plans, and strategies for developing mental health systems they simply will not happen (Langhaug et al., 2020). This requires push-in so that officials can educate themselves and others.
° Prepare to address common challenges (i.e., accessibility, acceptability, affordability, and stigma) that dissuade the use of mental health services. Articulate a country-level commitment to addressing these needs.
° Identify leaders from important sectors, such as public health, higher education institutions, technology, and the ministry of child and family who can serve as advocates.

Every health care program run by public health sectors and the minister of education (e.g., university teaching or training of health care workers) should establish a formal reciprocal relationship that includes a communication and data sharing plan. Reciprocal partnerships needs to explicitly detail a position of co-leadership and determine organizational practices that will instill trust and respect. New collaborations often fail when priorities compete and there is not a shared language to clarify intent and impact. As such, preparing processes to reconcile differences is important. The commitment to practical healthcare which similarly values the cultural and traditional differences noted among different sectors will allow for the candid discussions that can result in practical mental health outcomes for Ethiopians. For example, a predominately male healthcare workforce may not be beneficial to groups, such as women experiencing perinatal distress or mothers participating in their child's mental treatment.

Research tools and educational materials can be developed faster by establishing collaborative efforts with government officials (Zeleke et al., 2019). The intervention that promotes mental health in Ethiopia might follow from an approach aimed at the empowerment of specific groups (i.e., traditional healers, religious leaders, and herbalists) in the population because of the collective cultural practices and the reference to indigenous wisdom used by the general public. Likewise data-driven and evidence-based programs may result in good outcomes for those who will use it. Therefore, funding and support for developing these programs would be highly important for government officials to consider.

**2. Develop a Public Health Campaign to reduce stigma and focus on the collective health of society**. To prioritize the health of the next generation of Ethiopians, the mental health needs of children should be a high priority. Mental health and wellness needs to begin by addressing the mother's pregnancy and then the child's social-emotional development in the context of poverty and other prioritized stressors. Parent's need to understand how they can shape their child's development through Westernized practices that are bridged with traditional care. Such an effort needs to prioritize early intervention, as research consistently highlights its benefits in US (Blueprints for Healthy Youth Development, 2018) and may be promising for the diverse contexts (Breland-Noble et al., 2016). Given the focus on children's education, schools offer a venue for both identifying challenges and providing services.
° The gap between societal awareness and service opportunities could also be narrowed through a collaborative effort between the health and technology sectors. Although Ethiopia doesn't yet have an adequate pool of highly trained professionals to address all of the mental health issues, there is an accelerated growth in mobile and internet connectivity that could boost the development of electronic-mental health service provision.

Health professionals, organizational leaders, and community leaders have a responsibility to work collaboratively to mobilize, support, and empower mental health treatment. Therefore, they must be willing to undergo capacity building, training, engage in research, and develop a commitment to create enabling environments (Abayneh et al., 2020). With collaborative actions across government officials, community leaders, research, university professionals, health care professionals, medical professionals, and even individuals in the community, the mental health service system can be widely accepted and adapted to fit the needs of the individuals within the culture. Without working from a top-down approach, the stigma, limited education, and limited access to resources will continue. Therefore, this paper calls for action across multiple stakeholders to ensure the best support and resources related to mental health in Ethiopia. Recognizing these needs can make measurable improvements toward serving the next generation of children, and therefore improvement in societal mental health.

## Author Contributions

All authors listed have made a substantial, direct and intellectual contribution to the work, and approved it for publication.

## Conflict of Interest

The authors declare that the research was conducted in the absence of any commercial or financial relationships that could be construed as a potential conflict of interest.
